# Editorial: Autophagy and hypoxia-inducible factor in diabetes

**DOI:** 10.3389/fendo.2023.1349432

**Published:** 2023-12-18

**Authors:** Jing Guo, Xuefei Tian, Huafeng Liu, Wei Jing Liu

**Affiliations:** ^1^ Institute of Basic Research in Clinical Medicine, China Academy of Chinese Medical Sciences, Beijing, China; ^2^ Section of Nephrology, Department of Internal Medicine, Yale School of Medicine, CT, New Haven, United States; ^3^ Key Laboratory of Prevention and Management of Chronic Kidney Disease of Zhanjiang City, Affiliated Hospital of Guangdong Medical University, Zhanjiang, China; ^4^ Renal Research Institution of Beijing University of Chinese Medicine, and Key Laboratory of Chinese Internal Medicine of Ministry of Education and Beijing, Dongzhimen Hospital Affiliated to Beijing University of Chinese Medicine, Beijing, China

**Keywords:** diabetes mellitus, autophagy, natural products, diabetic kidney disease, HMGB1

Diabetes mellitus (DM), as a chronic non-communicable disease, continues to be one of the largest public health problems around the world, leading to reduced quality of life and higher premature mortality. The World Health Organization (WHO) reports a staggering increase in the prevalence of type 2 diabetes over the past decades, affecting countries across all income levels. Presently, an estimated 422 million individuals worldwide live with DM, resulting in 1.5 million deaths directly attributed to diabetes annually.The prevalence of DM keeps steadily and the number is projected to reach 643 million by 2030, and 783 million by 2045. Beyond the issue of elevated glucose levels, individuals with DM grapple with associated complications that manifest concurrently with the diagnosis. While there have been advancements in medications aimed at not only managing blood glucose levels but also providing multi-organ protection, exemplified by SGLT2 inhibitors, these medications are not devoid of side effects, and organ injuries often persist with limited reversibility. In light of these challenges, the identification and elucidation of putative underlying mechanisms of DM and its complications are crucial,which contribute to the identification of potential therapeutic targets for future interventions.

Currently, dysregulation of autophagy and the involvement of hypoxia have been recognized as additional contributors to the pathogenesis of diabetes and resultant organ injuries. However, the specific mechanisms underlying these phenomena remain elusive, given the presence of conflicting and controversial results in reported studies. This complexity is attributed to the intricate pathological milieu of diabetes, encompassing factors such as the reactive oxygen species environment, excessive formation of advanced glycation end products, and hyperinsulinemia.The Research Topic aims to underscore the latest advancements in comprehensively and deeply unraveling the molecular mechanisms of autophagy and hypoxia-inducible factors in diabetes. Additionally, the intention is to explore potential therapeutic avenues in addressing these intricate pathways and their implications for diabetes management.

Accumulating evidence suggests that autophagy defects are clearly linked with β-cell dysfunction in the context of type 2 diabetes. Cui and Li explored bioinformatics analysis to identify autophagy related genes of pancreatic β cells, which may serve as potentical biomarkers for type 2 DM. Thirty differentially expressed autophagy related genes(DEARGs) enriched in autophagy- and mitopahgy-related pathways were discovered. The top ten DEARGs were screened by calculating network topology parameters, including GAPDH, ITPR1, EIF2AK3, FOXO3, HSPA5, RB1CC1, LAMP2, GABARAPL2, RAB7A, and WIPI1. Two pancreatic β-cell types were used to construct experiment validation. The results showed that five autophagy-related genes, including EIF2AK3, GABARAPL2, HSPA5, LAMP2, and RB1CC1 were both differentially expressed in both NE2SY and INS-1 cells treated with streptozotocin(STZ). Moreover, overexpression EIF2AK3 or RB1CC1 induced the increased expression of GABARAPL2, HSPA5 and LAMP2, as well as improved cell viability and insulin secretion. This study has the potential to provide novel insights into the role of DEARGs in type 2 DM and identify potential biomarkers that can be targeted for therapeutic interventions.

The protein High-mobility group box1 (HMGB1) is recognized for its role in governing gene transcription and cellular processes through its interaction with DNA or chromatin, facilitated by the receptor for advanced glycation end-products (RAGE) and toll-like receptor 4 (TLR4). HMGB1 plays an important role in DM, including inducing autophagy. Yang et al. comprehensively reviewed literature and summarized regulatory relationship between HMGB1 and autophagy in DM and its complications. Both HMGB1 and autophagy participate in maintaining the homeostasis of pancreatic β cells, whose dysfunction leading to insulin resistance, DM and development of diabetic complications. Impressively, the interaction between HMGB1 and autophagy is bidirectional. HMGB1 plays a pivotal role in autophagy induction and can activate autophagy in multiple ways depending on its location. Autophagy regulated the production, secretion and degradation of HMGB1. Meanwhile, in the context of summarizing the mechanism of action of hypoglycemic drugs, both HMGB1 and autophagy emerge as promising therapeutic targets. However, in DM and diabetic complications, the pathological mechanisms of autophagy dysfunction, abnormal HMGB1 expression, and induced injury remain largely unexplored.

Diabetic kidney disease(DKD), as one of the microvascular complications, is the leading cause of kidney failure and increase the risk of cardiovascular diseases. Natural products, including Chinese herbal medicine and bioactive compounds, have been demonstrated as promising therapeutic candidates for DKD. Liu et al. provided a comprehensive summary highlighting the dysregulation or insufficiency of autophagy in renal cells. Their systematic review focused on elucidating the mechanism of autophagy modulation in DKD by various Chinese herb compound preparations, single herbs, and active compounds aiming to offer novel drug candidates for the clinical treatment of DKD.Consistently, Liu et al. conducted a thorough review on the dysregulation of autophagy in Diabetic Kidney Disease (DKD). Their focus extended to the robust exploration of autophagy regulatory pathways, mitophagy and lipophagy, as well as autophagy regulation within renal resident cells and renal macrophages. Noteworthy is their comprehensive summary of the potential applications and mechanisms associated with certain well-known natural polyphenols, such as resveratrol, curcumin, puerarin, among others, as autophagy regulators in DKD, contributing to a deeper understanding of the mechanisms underlying natural polyphenols’ efficacy in treating DKD and to foster advancements in their practical applications.

The Research Topic has successfully compiled valuable studies and contributions focusing on autophagy, emphasizing its promising therapeutic potential in DM and associated complications. Regrettably, no pertinent studies on hypoxia and hypoxia-inducing factors in diabetes and its complications were included in our Research Topic. We anticipate that this research area will serve as an inspiration for more in-depth investigations into the mechanisms and therapeutic strategies involving both autophagy and hypoxia-inducing factors in the future.

## Author contributions

JG: Writing – original draft. XT: Writing – review & editing. HL: Writing – review & editing. WL: Writing – review & editing.

